# Selective Capture and Identification of Methicillin-Resistant *Staphylococcus aureus* by Combining Aptamer-Modified Magnetic Nanoparticles and Mass Spectrometry

**DOI:** 10.3390/ijms22126571

**Published:** 2021-06-18

**Authors:** Yu-Chen Liu, Katragunta Kumar, Cheng-Hsiu Wu, Kai-Chih Chang, Cheng-Kang Chiang, Yen-Peng Ho

**Affiliations:** 1 Department of Chemistry, National Dong Hwa University, Hualien 974, Taiwan; yu024527@gmail.com (Y.-C.L.); kumarkatragunta@gmail.com (K.K.); 410312048@gms.ndhu.edu.tw (C.-H.W.); ckchiang@gms.ndhu.edu.tw (C.-K.C.); 2 Department of Laboratory Medicine and Biotechnology, Tzu Chi University, Hualien 970, Taiwan; kaichih@mail.tcu.edu.tw

**Keywords:** methicillin-resistant *Staphylococcus aureus*, aptamer, magnetic nanoparticles, antibiotic resistance, MALDI-MS

## Abstract

A nucleic acid aptamer that specifically recognizes methicillin-resistant *Staphylococcus aureus* (MRSA) has been immobilized on magnetic nanoparticles to capture the target bacteria prior to mass spectrometry analysis. After the MRSA species were captured, they were further eluted from the nanoparticles and identified using matrix-assisted laser desorption ionization mass spectrometry (MALDI-MS). The combination of aptamer-based capture/enrichment and MS analysis of microorganisms took advantage of the selectivity of both techniques and should enhance the accuracy of MRSA identification. The capture and elution efficiencies for MRSA were optimized by examining factors such as incubation time, temperature, and elution solvents. The aptamer-modified magnetic nanoparticles showed a capture rate of more than 90% under the optimized condition, whereas the capture rates were less than 11% for non-target bacteria. The as-prepared nanoparticles exhibited only a 5% decrease in the capture rate and a 9% decrease in the elution rate after 10 successive cycles of utilization. Most importantly, the aptamer-modified nanoparticles revealed an excellent selectivity towards MRSA in bacterial mixtures. The capture of MRSA at a concentration of 10^2^ CFU/mL remained at a good percentage of 82% even when the other two species were at 10^4^ times higher concentration (10^6^ CFU/mL). Further, the eluted MRSA bacteria were successfully identified using MALDI mass spectrometry.

## 1. Introduction

Antibiotic resistance has become one of the biggest threats to global health and food security. Many infections are difficult to treat as the antibiotics become less effective. The emergence and spread of the resistance are accelerated by the misuse and overuse of antibiotics. *Staphylococcus* *aureus* is known for its tendency to develop resistance to various classes of antibiotics over time. Methicillin-resistant *Staphylococcus aureus* infections often happens in hospitals, particularly among immunocompromised patients. The resistance of MRSA is because of the nonnative gene, *mecA,* that encodes a penicillin-binding protein 2a (PBP2a). The protein catalyzes transpeptidation reaction for cell wall synthesis and peptidoglycan cross-linking in the presence of antibiotics [1]. Because MRSA is one of the most often encountered bacteria in clinical practice, it has been corresponding to higher healthcare costs than the methicillin-susceptible *S. aureus*. Identification of MRSA in clinical samples in a fast, accurate, and inexpensive manner would benefit both the testing lab and healthcare providers.

Many conventional methods have been developed to detect MRSA. Culture-based methods such as the disk diffusion test and microdilution method measure the minimum inhibitory concentration of antibiotics [2]. DNA-based methods like PCR target the *mecA* gene [3,4]. Immunological methods such as ELISA have been employed to detect the presence of the penicillin-binding protein [5]. These methods have limitations. For instance, the culture-based methods are time-consuming. The immunological methods require antibodies to capture or detect targets. Aptamers are single-stranded oligonucleotide which can fold into a definable three-dimensional structure and bind to specific proteins or metabolites [6,7]. In general, these specific aptamers are selected using systematic evaluation of ligands by exponential enrichment (SELEX) [8,9]. Aptamers are similar to antibodies in functionalities but do not elicit immunogenicity. They are easy to be reproduced by chemical synthesis and relatively small in size. Furthermore, aptamers have higher stability and longer shelf life in comparison to antibodies [10,11]. Therefore, the aptamers are quite suitable for bacterial analysis in clinical laboratories [12,13].

Nanomaterials such as functionalized magnetic nanoparticles (MNPs) have been widely used in the enrichment of targeted analytes during sample preparation processes [14]. Nanoparticles with various functionalities have been designed to purify and concentrate bacteria. The nanomaterials conjugated with aptamers facilitate recognition, rapid enrichment, and separation of targets. Aptamer-based affinity approaches have been used in the analysis of peptides and proteins in bacterial cells and human samples [15,16,17,18]. A recent report revealed the application of enzyme-linked DNA aptamer to the detection of outer membrane vesicles in Gram-negative bacterial cells for the early diagnosis of bacterial infections [17].

Aptamer-based approaches [19,20] have been coupled to many readout systems such as colorimetry, fluorescence, and electrochemistry. Although each of these methods has its strength, such as simplicity or sensitivity, nonspecific interactions of aptamers with interferences in the samples pose a major challenge to the accuracy of biosensing approaches.

Mass spectrometry (MS) is a useful tool for microbial analysis [21,22,23,24] because of its rapidness, minimal sample preparation, and high selectivity. Currently, matrix-assisted laser desorption/ionization and electrospray ionization (ESI) are two main ionization techniques for MS analysis of biomolecules. Specific spectral profiles could be obtained from ESI- and MALDI-MS analysis of the proteins extracted from bacteria. These protein patterns could be utilized as bacteria fingerprints for species identification [25,26,27]. Intact cell mass spectrometry was also developed to identify microorganisms without sample pre-treatment [28,29]. Alternatively, the extracted proteins from bacteria cells may be digested into peptides with specific enzymes. Digests are subjected to liquid chromatography-ESI-MS analysis, and the proteins associated with certain bacterial species may be identified through tandem mass spectrometry [30,31,32]. Currently, most published reports of the rapid mass spectrometric analysis of microorganisms without chromatographic separation are based on MALDI-MS because of its speed and simplicity [33,34,35,36,37]. The combination of aptamer-based capture/enrichment and MS analysis of microorganisms takes advantage of the selectivity of both techniques and should enhance the accuracy of microbial identification. A peptide aptamer that recognizes both SA and MRSA has been immobilized on gold nanoparticles to capture the bacteria prior to surface-assisted laser desorption ionization-MS analysis [38,39]. The present study aims to develop an affinity mass spectrometric method for selective analysis of MRSA. Reusable magnetic nanoparticles modified with nucleic acid aptamers were used to selectively capture and enrich MRSA bacteria. The MRSA species were further confirmed by MALDI-MS.

## 2. Results and Discussion

In the present study, the selectivity of aptamer-modified magnetic nanoparticles towards MRSA bacteria was investigated. The magnetic iron oxide core was coated with silica via the sol-gel method. Then, the particle surface was modified with carboxylic groups through a reaction with polyacrylic acid (PA) to form PA-modified magnetic silica nanoparticles (PAMNPs). The aptamer was conjugated with PAMNPs through a coupling reaction between the amino group in the aptamer and the carboxyl group in the particles.

### 2.1. IR Spectra of PAMNPs

**Figure 1:**
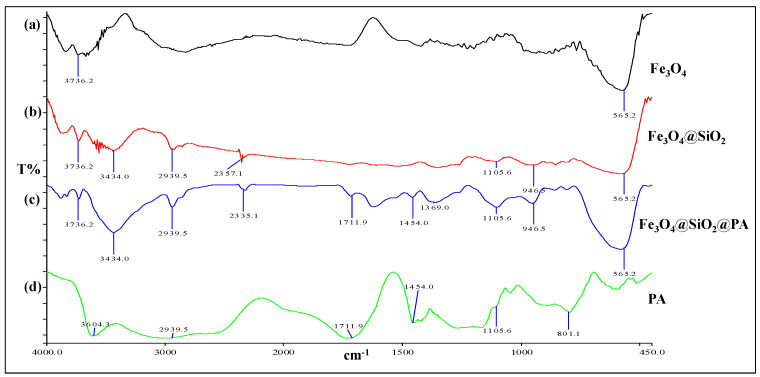
FTIR spectra of (**a**) Fe_3_O_4_ magnetic nanoparticle, (**b**) Fe_3_O_4_@SiO_2_, (**c**) Fe_3_O_4_@SiO_2_@PA, and (**d**) polyacrylic acid (PA).

The chemical modifications were confirmed by FTIR analyses. Figure 1 illustrates the FTIR spectra of Fe_3_O_4_ nanoparticles, Fe_3_O_4_@SiO_2_, Fe_3_O_4_@SiO_2_@PA, and PA. The IR spectrum of Fe_3_O_4_ nanoparticles shows absorption bands at 3736.2 cm^−1^ due to -OH stretching and at 565.2 cm^−1^ due to Fe-O stretching (Figure 1a). Figure 1b shows the Fe_3_O_4_@SiO_2_ IR spectrum indicating bands at 1105.6 cm^−1^ and 565.2 cm^−1^ due to Si-O-Si asymmetrical stretching and Fe-O stretching, respectively. The IR spectrum of Fe_3_O_4_@SiO_2_@PA (Figure 1c) reveals multiple bands at 565.2 cm^−1^, 1105.6 cm^−1^, 1454.0 cm^−1^, and 1711.9 cm^−1^ corresponding to Fe-O stretching, CO asymmetrical stretching, C=C stretching, and C=O stretching, respectively. Figure 1d illustrates the IR spectrum of PA with absorption bands at 1105.6 cm^−1^, 1454.0 cm^−1^, and 1711.9 cm^−1^ arising from the -CO asymmetrical stretching, C=C stretching, and C=O stretching, respectively. The results indicate the successful functionalization of PA onto the surface of the magnetic silica nanoparticles.

### 2.2. Conjugation of Aptamers with PAMNPs

**Figure 2:**
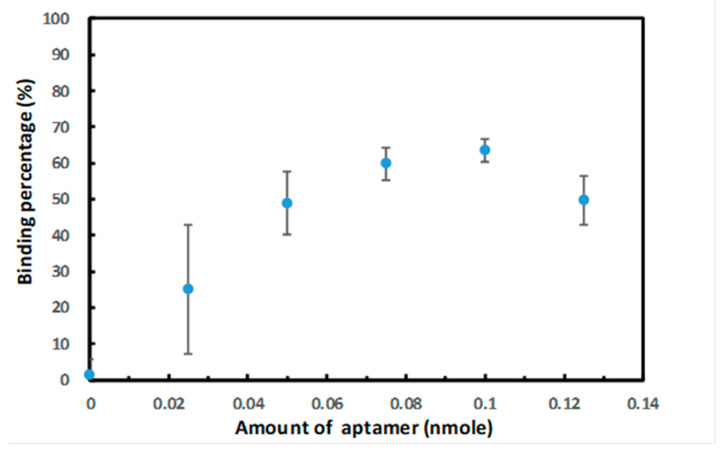
Plot of binding percentage vs. the amount of aptamers conjugated with 1 mg of PAMNPs.

We further evaluated the efficiency of covalent binding between the carboxyl groups on PAMNPs and the amino groups on DNA aptamers. The reaction time and the amounts of aptamers used for the conjugation experiments were optimized. The optimum reaction time was determined based on the binding percentage. The binding percentage between the aptamer and PAMNPs was calculated by measuring the UV absorption of unbound aptamers in the supernatant after the binding reaction (Figure S1). The percentage of aptamers bound to the particles increased gradually with increasing reaction time. The binding percentage increased 10% from 1 h to 2 h of reaction, but the increase slowed down between 3 h and 4 h. The binding percentage reached 66.8% after 4 h of reaction. Considering the time efficiency, 3 h of reaction time with a binding percentage of 61.1% was used for the conjugation reaction. We evaluated the amount of aptamer bound with 1 mg of PAMNPs after the mixture was incubated for 3 h. The binding percentages were determined at various amounts of aptamer (Figure 2). The results suggest that 0.1 nmole of the aptamer under 3 h of reaction gave the best binding rate (63%). When the amount of aptamer was increased to 0.125 nmole, the binding percentage was decreased to 50%, indicating saturation of the binding sites on PAMNPs for aptamers.

### 2.3. Bacterial Capture Efficiency of PAMNP-Aptamer

**Figure 3:**
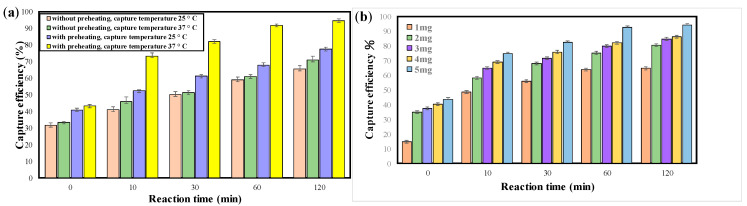
Optimization of reaction conditions for the capture experiments using PAMNP-aptamer: (**a**) capture efficiency (at 25 °C or 37 °C) of MRSA bacteria (10^7^ CFU/mL) with and without preheating (100 °C, 5 min) of PAMNP-aptamer; (**b**) capture of MRSA bacteria at different reaction times from 0 to 2 h with varying amount of PAMNP-aptamer (1–5 mg).

The magnetic nanoparticles modified with aptamers were used to capture MRSA bacteria (10^7^ CFU/mL). To enhance the capture capability of PAMNP-aptamer, preheating of the nanoparticles was found to be very critical. The PAMNP-aptamer was heated at 100 °C for 5 min and cooled down to 25 °C or 37 °C prior to the capture processes. As shown in Figure 3a, the capture efficiency with preheating was better than that without preheating at any given reaction times. The number of bacteria captured was determined based on a calibration curve (Figure S2) established from the fluorescence intensity vs. the concentration of fluorescein isothiocyanate (FITC)-labeled bacteria. The calibration showed good linearity through the concentration range between 10 and 10^7^ CFU/mL of MRSA.

There might be incorrect adhesion and entanglement between some of the aptamers on the surface of the nanoparticles without preheating. Heating to high temperature and cooling down slowly to appropriate temperature caused the aptamers to denature and reform into a native tertiary structure that was a suitable conformation to interact with the target bacteria. If we rapidly cooled down the aptamers in an ice bath, the capture efficiency was less than 5% (data not shown) because the aptamers could not assume the native conformation. The results proved that preheating and slow cooling facilitated the formation of the native structure of the aptamers and, therefore, enhanced the capture efficiency. The capture efficiency was also compared between 25 °C and 37 °C (Figure 3a), revealing a better capture performance at 37 °C than at 25 °C. The result is expected because the aptamer should be more stable in physiological temperature (37 °C).

The amount of the modified nanoparticles (1–5 mg) was optimized for MRSA capture in 1 mL of PBS (pH 7.5, 37 °C) at different reaction times from 0 to 2 h. The results indicate that the capture efficiency increases with the increasing reaction time and amounts of PAMNP-aptamer. The capture efficiency at 1 h and 2 h reaction time reached 92 and 94%, respectively, when 5 mg of PAMNP-aptamer was used (Figure 3b). An increase of nanoparticles to 10 mg slightly improved the capture efficiency to 96% (Figure S3), but a further increase of the particles up to 25 mg did not improve the capture rate of MRSA. The reaction time of 1 h and particle amounts of 5 mg was used in this study.

**Figure 4:**
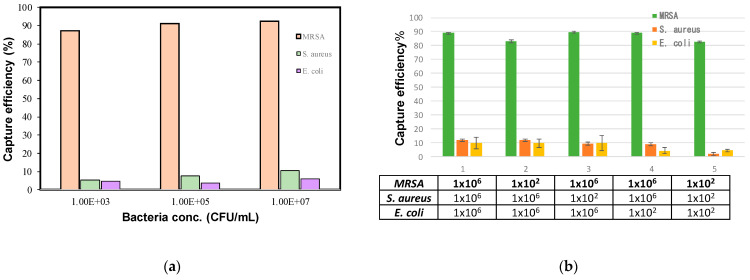
(**a**) Capture efficiency of different FITC-labeled bacteria (MRSA, *S. aureus*, *E. coli*) at different concentrations (10^3^, 10^5^, 10^7^ CFU/mL). (**b**) Selectivity of aptamer-modified nanoparticles examined in bacteria mixtures containing various ratios of the three species.

The selectivity of the aptamer-modified nanoparticles towards MRSA was investigated by determining the capture rates of MRSA and two other non-target bacterial species, non-resistant *S. aureus* and *E. coli,* at concentrations of 10^3^, 10^5^, and 10^7^ CFU/mL (Figure 4a). Under the optimized conditions, the capture rates reached 87%, 91%, and 92%, respectively, for the FITC-labelled MRSA at 10^3^, 10^5^, and 10^7^ CFU/mL. The capture rates for non-resistant *S. aureus* and *E. coli* at 10^3^ CFU/mL were 5% and 4%, respectively. The capture rates for the non-target bacteria at 10^5^ and 10^7^ CFU/mL were all less than 11%. The results suggest that the PAMNP-aptamer nanoparticles have very good selectivity towards MRSA.

Further, the selectivity of the aptamer-modified nanoparticles was examined in bacteria mixtures containing various ratios of the three species. In each experiment, only one of the bacterial species was labeled with the FITC dye so that the capture rate of the labeled species could be determined by the fluorescence measurement. The samples were prepared by mixing MRSA, *S. aureus*, and *E. coli* at concentrations of 10^2^ or 10^6^ CFU/mL. As shown in Figure 4b, the capture rate for MRSA is 89% when the three species were mixed at each concentration of 10^6^ CFU/mL. The capture rate for MRSA was 83% when the three species were mixed with each concentration at 10^2^ CFU/mL. The capture rate of MRSA at a concentration of 10^2^ CFU/mL was 82%, even when the other two species were at a much higher concentration of 10^6^ CFU/mL. It shows that a relatively large amount of non-target bacteria does not have considerable interference on the MRSA capture efficiency.

### 2.4. Optimization of Elution Efficiency

**Figure 5:**
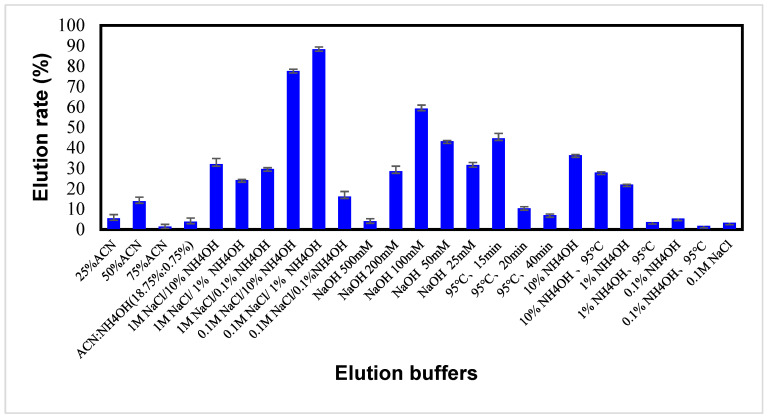
Elution rates of MRSA using different elution solvents.

To identify the bacteria, the captured bacteria can be eluted for fluorescence or mass spectrometric analysis. Therefore, the elution efficiency was evaluated based on elution conditions, including elution solvents, salt (NaCl) content, temperature, and pH. Figure 5 shows the elution efficiency using various elution reagents and conditions. Use of the NaOH solution as the elution solvent yielded a moderate elution percentage ranging from 30 to 60% if the NaOH concentration was less than 0.5 M. The organic solution of acetonitrile in methanol was not an effective elution solvent. The eluates obtained from both the NaOH and the organic solution showed a yellowish-brown color, and the magnetic nanoparticles could not be reused, indicating damage to the particles. Ammonium hydroxide (NH_4_OH) mixed with the sodium chloride solution seems to be an efficient eluent. The 1% NH_4_OH/0.1 M NaCl gave an elution rate of 92%. Interestingly, the NH_4_OH or NaCl solution alone was not an efficient elution solvent. The NH_4_OH solution only yielded an elution rate of less than 40%, while 0.1 M NaCl alone gave less than 5%. The ammonium hydroxide may disrupt the interaction between the aptamer and bacteria, and the sodium chloride is believed to bind with the aptamer structure through ionic interaction after the disruption. The ionic interaction might prevent the reattachment of bacteria to the aptamers. Therefore, the combination of ammonium hydroxide and sodium chloride led to the best elution effect.

**Figure 6:**
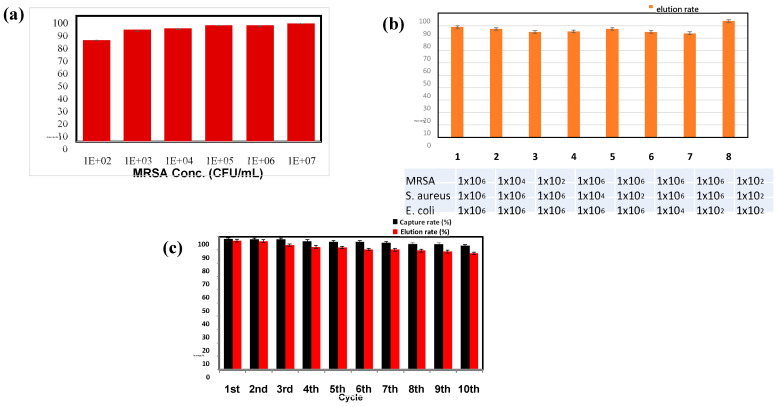
(**a**) Elution efficiency of MRSA bacteria at different concentrations (10^2^–10^7^ CFU/mL). (**b**) Elution of captured MRSA from PAMNP-aptamer in samples containing bacteria mixtures of three species mixed at different concentrations. (**c**) Capture and elution percentages for the PAMNP-aptamer approach during 10 consecutive cycles of experiments.

The elution solvent (0.1 M NaCl/1% NH_4_OH) was used to elute MRSA after the bacteria (10^2^–10^7^ CFU/mL) were captured with 5 mg of aptamer-modified nanoparticles. The elution rate was 80% when the bacterial concentration was 10^2^ CFU/mL, as shown in Figure 6a. All the elution rates reached 89% or higher at bacterial concentrations ranging from 10^3^ to 10^7^ CFU/mL. The elution of MRSA was also examined in bacteria mixtures containing various ratios of three species (Figure 6b). In each experiment, only MRSA was labeled with the FITC dye so that the elution rate of the labeled species could be determined by the fluorescence measurement. The samples were prepared by mixing MRSA, *S. aureus*, and *E. coli* at concentrations of 10^2^, 10^4^, or 10^6^ CFU/mL. The samples were subjected to capture and elution processes. The elution rate of MRSA was 89% when the three species was at the same concentration of 10^6^ CFU/mL. The elution rate for MRSA was as good as 85% even when the target MRSA was at a lower concentration of 10^2^ CFU/mL and the other two bacteria were at 10^4^ times higher concentration (10^6^ CFU/mL). When all the three bacteria species were mixed at equal concentrations of 10^2^ CFU/mL, the elution rate was increased to 94%. The overall results indicate that the modified nanoparticle may recover a low concentration of MRSA from sample mixtures.

The reusability of the aptamer-based nanoparticles was evaluated by performing 10 consecutive cycles of capture and elution of MRSA bacteria. The capture and elution rates are shown in Figure 6c. During the multiple cycles of reuse, there was no substantial decrease in the capture and elution rate of MRSA (10^7^ CFU/mL). In the first cycle, APMNP-aptamer showed 98% of capture rate and 97% of elution rate, while in the tenth consecutive cycle, the capture rate and elution rate were 93% and 88%, respectively. It was only a 5% decrease in the capture rate and a 9% decrease in the elution rate after 10 cycles, implying good reusability of the aptamer-modified nanoparticles.

### 2.5. Mass Spectrometric Analysis

**Figure 7:**
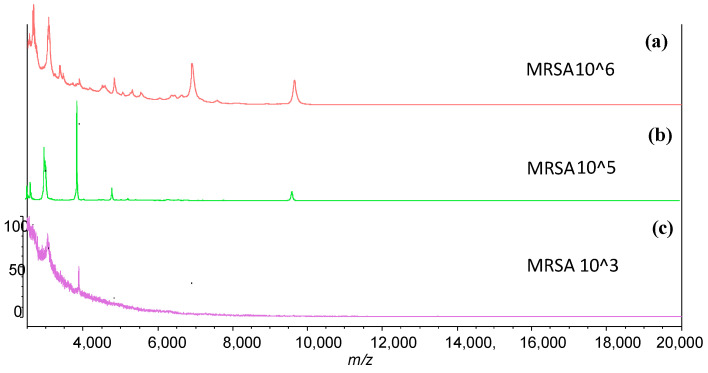
MALDI spectra of MRSA (**a**) obtained from a pure culture at a concentration of 10^6^ CFU/mL, (**b**) captured with PAMNP-aptamer in the bacterial mixture containing MRSA and two other species (*S. aureus* and *E. coli*) at the same concentrations of 10^5^ CFU/mL, and (**c**) captured with PAMNP-aptamer in the bacterial mixture containing the three species at the same concentrations of 10^3^ CFU/mL.

MALDI-MS was applied to the analysis of MRSA in a bacterial mixture containing MRSA, *S. aureus*, and *E. coli*. After the MRSA bacteria were captured by APMNP-aptamer, the eluted bacteria were analyzed using MALDI-MS. Figure 7a shows a MALDI spectrum of MRSA at a concentration of 10^6^ CFU/mL obtained from a pure bacterial culture. To identify the protein signals observed in the spectrum, protein digestion and LC-MS analysis should be carried out. Herein, the peaks observed in the MALDI spectrum were used as fingerprint signals. Although the identities of these peaks were not known, they provide extra identification data of MRSA after selectively captured by APMNP-aptamer. Figure 7b displays the MALDI spectrum of MRSA at a concentration of 10^5^ CFU/mL obtained from the bacterial mixture containing two other species (*S. aureus* and *E. coli*) at the same concentrations of 10^5^ CFU/mL. Although there were not as many peaks as those in the spectrum of pure MRSA, several characteristic peaks associated with MRSA were observed. When the three bacterial species were mixed at a concentration of 10^3^ CFU/mL, respectively, two characteristic peaks corresponding to the MRSA were identified from the eluted sample (Figure 7c). The concentration at 10^3^ CFU/mL appears to be the limit of detection for the current MALDI mass spectrometer because we did not detect useful signals from the bacterial mixture at a concentration of 10^2^ CFU/mL. Nevertheless, the selectivity of APMNP-aptamer and mass spectrometry should raise the confidence in MRSA identification.

## 3. Materials and Methods

### 3.1. Materials and Reagents

Iron(II) chloride tetrahydrate (99%) and Iron(III) chloride hexahydrate (99%) were obtained from Acros (Morris Plains, NJ, USA). Poly (acrylic acid) (MW ~ 100,000) solution was purchased from Sigma-Aldrich (Steinheim, Germany). Hydrochloric acid (36.5%) was purchased from J.T. Baker (Phillipsburg, PA, USA). Ammonia hydroxide solution (25%) was obtained from Wako Pure Chemical Industries (Osaka, Japan). Tetraethyl orthosilicate (TEOS) was obtained from Fluka (Seelze, Germany). Acetonitrile, methanol, ethanol (99.5%), 1-Ethyl-3-(3-dimethylamino- propyl) carbodiimide (EDC), 2-(N-morpholino) ethanesulfonic acid (MES), and fluorescein isothiocyanate (FITC) were obtained from Sigma Aldrich (St. Louis, MO, USA). cDNA aptamers were purchased from Scientific Biotech. Corp. (Taipei, Taiwan). The 5′ amino-modified aptamer was a single-stranded DNA with a gene sequence [40] shown below:

5′ATCCAGACGTGACGCAGCATGCGGTTGGTTGCGGTTGGGCATGATGTATTTCTGTGTGGACACGGTGGCTTAGTA-3′.

### 3.2. Instruments

Fourier-transform infrared (FTIR) spectra were recorded on a Spectrum One System (PerkinElmer, Waltham, MA, USA). The KBr pellet method was applied to the analysis of nanoparticle powder by mixing the sample powder (ca. 1% of the KBr amount) with KBr. UV-Vis absorption spectra were measured on a UV-Vis spectrophotometer (Model U-3900, Hitachi, Japan). Fluorescence spectra were recorded using a Multi-Mode Microplate Reader (Model SpectraMax iD3, Molecular Devices LLC, San Jose, CA, USA).

Mass spectra were acquired using an AXIMA Performance MALDI TOF-TOF mass spectrometer (Shimadzu Corporation, Kyoto, Japan) equipped with a 337 nm nitrogen laser. Ion source, lens, and linear detector voltages were set at 20.0, 6.0, and 2.8 kV, respectively. MALDI-TOF mass spectra were acquired in the positive linear mode with 200 shots from random positions on the same sample spot. Spectra were acquired as the sum of the laser shots. The bacteria were suspended in aqueous 70% (*v/v*) formic acid (FA). The saturated matrix solution was prepared by dissolving α-cyano-4-hydroxycinnamic acid (CHCA) into a solution of 50% acetonitrile/water containing 0.1% formic acid. A 1 µL aliquot of bacteria sample in 70% FA mixed with an equal volume of CHCA matrix solution was spotted on a target plate and allowed to air-dry before MALDI-MS analysis.

### 3.3. Bacterial Samples

The clinical bacterial samples used in this study were obtained from the Dr. Kai-Chih Chang laboratory. *Escherichia coli* (*E. coli*), *Staphylococcus aureus*, and MRSA were cultured separately in 3 mL of LB agar medium and incubated at 37 °C with gentle shaking for 12 h. Then, the bacteria were quantified by measuring the optical density at 600 nm using a UV/Vis spectrometer and washed several times with a PBS buffer for further use. In bacterial labeling experiments, bacterial cells were labeled with fluorescein isothiocyanate. The bacteria suspended in 1 mL of PBS (pH 7.4) were mixed with 5 mg of FITC and stirred on ice for 4 h. The FITC-labeled bacteria were collected by centrifugation and washed four times with PBS. The collected bacteria were stored in a PBS buffer.

### 3.4. Synthesis of Magnetic Fe_3_O_4_ Nanoparticles Modified with Carboxylic Group (Fe_3_O_4_@SiO_2_@PA)

Magnetic iron oxide nanoparticles were prepared via a co-precipitation method [14]. FeCl_2_·4H_2_O (4 g) and FeCl_3_·6H_2_O (10.8 g) were dissolved in 25 mL of 2 M HCl and the solution was deaerated by nitrogen purging. Then, 60 mL of 25% aqueous ammonia solution was slowly added to the solution with stirring and nitrogen purging. The reaction was continued for 30 min at room temperature and the resulting iron oxide magnetic nanoparticles were rinsed three times with 50 mL of deionized water and two times with 40 mL of ethanol. The particles were resuspended in 160 mL of ethanol. After the suspension was sonicated for 1 h, 15 mL of 25% ammonia solution, 12.5 mL of deionized water and 250 µL tetraethyl orthosillicate were sequentially added to the suspension. The reaction was carried out at 40 °C under nitrogen for 2 h. To strengthen the structure, the synthesized SiO_2_-coated iron oxide particles (Fe_3_O_4_@SiO_2_) were heated under reflux at 60 °C for 12 h. The resulting particles were washed with ethanol and resuspended in 100 mL ethanol. The Fe_3_O_4_@SiO_2_ suspension was mixed with an aqueous polyacrylic acid solution (100 mg/mL), and the mixture was stirred at room temperature for 8 h. The prepared Fe_3_O_4_@SiO_2_@PA particles were rinsed several times with deionized water and stored at 4 °C.

### 3.5. Synthesis of Aptamer-Modified Fe_3_O_4_@SiO_2_@PA Magnetic Nanoparticles (PAMNP-Aptamer)

The Fe3O4@SiO2@PA particles were washed with 100 µL of MES buffer and then mixed with 1.5 mL of MES and 0.6 mg of EDC. After one hour of reaction, 0.1 nmole of 5′ amino-modified aptamers were added to the suspension and the reaction was allowed to proceed at 37 °C for 3.5 h. The aptamer/magnetic nanoparticle conjugates (PAMNP-aptamer) were collected using a magnet and washed thoroughly with 0.1 M PBS. The unbound aptamers in the supernatant were determined by measuring the UV absorption at 260 nm. The binding rate of the aptamer with the nanoparticles was calculated based on the difference between the UV absorption of aptamer before (A_1_) and after (A_2_) the conjugation experiments: ((A_1_ − A_2_) × 100)/A_1_ (%).

### 3.6. Capture and Elution of Bacteria

The PAMNP-aptamer conjugates were heated at 100 °C for 5 min to denature the DNA aptamer and cooled down to 25 or 37 °C prior to the capture of target bacteria. Equal volumes (500 μL) of the modified nanoparticles and bacteria in 0.1 M PBS were mixed using a blood mixer under various conditions. After the capture reaction, the magnetic nanoparticles were separated from the supernatant by a magnet. The magnetic nanoparticles were washed three times with 200 μL PBS to remove unbound bacteria. The supernatants were combined and the fluorescence was measured at 530 nm under 480 nm irradiation. The bacteria concentrations of the original sample before capture and the supernatant after capture were quantified using a calibration curve, which was established based on the fluorescence intensities vs. bacteria concentrations. The number of bacteria bound with PAMNP-aptamer were determined from the difference in bacterial number between the original sample (total bacteria) and supernatant (uncaptured bacteria). The bacterial capture rate was calculated from the bacterial number bound with the particles divided by the number in original bacteria samples. The bound bacteria were eluted from the magnetic particles by mixing the bacteria-particle conjugates with 200 μL of elution solvents under vortex for 30 s. The elution percentage was determined by the ratio between the number of bacteria in eluate and the number of bound bacteria.

## 4. Conclusions

In summary, we describe a highly specific and robust method for capturing and analyzing methicillin-resistant *Staphylococcus aureus*. This method employed synthetic magnetic nanoparticles modified with nucleic acid aptamers to capture MRSA. The captured bacteria were eluted from the nanoparticles and analyzed by mass spectrometry. The aptamer-modified nanoparticles are easy to synthesize, easy to collect, and have high specificity. The aptamer-modified nanoparticles showed good selectivity towards MRSA bacteria with a capture rate of more than 90% under optimized conditions, whereas for non-resistant bacteria, the capture rates were less than 11%. The as-prepared nanoparticles exhibited only 5% decrease in the capture rate and 9% decrease in the elution rate after 10 successive cycles of utilization. More importantly, the eluted MRSA bacteria may be identified using MALDI mass spectrometry. The orthogonal mass spectrometric analysis provides further confirmation of microorganisms after a very selective capture of the target species.

## Data Availability

Not applicable.

## Supplementary Materials

The following are available online at https://www.mdpi.com/article/10.3390/ijms22126571/s1.

## References

[1] Peacock S.J., Paterson G.K. (2015). Mechanisms of methicillin resistance in *Staphylococcus aureus*. Annu. Rev. Biochem..

[2] Balouiri M., Sadiki M., Ibnsouda S.K. (2016). Methods for in vitro evaluating antimicrobial activity: A review. J. Pharm. Anal..

[3] Barski P., Piechowicz L., Galinski J., Kur J. (1996). Rapid assay for detection of methicillin-resistant *Staphylococcus aureus* using multiplex PCR. Mol. Cell Probe.

[4] Nair D., Shashindran N., Kumar A., Vinodh V., Biswas L., Biswas R. (2021). Comparison of phenotypic MRSA detection methods with PCR for mecA gene in the background of emergence of oxacillin-susceptible MRSA. Microb. Drug Resist..

[5] Peng J., Cheng G.Y., Huang L.L., Wang Y.L., Hao H.H., Peng D.P., Liu Z.L., Yuan Z.H. (2013). Development of a direct ELISA based on carboxy-terminal of penicillin-binding protein BlaR for the detection of beta-lactam antibiotics in foods. Anal. Bioanal Chem..

[6] Cataldo R., De Nunzio G., Millithaler J.F., Alfinito E. (2020). Aptamers which Target Proteins: What proteotronics suggests to pharmaceutics. Curr. Pharm. Des..

[7] Lee B.H., Kwon Y.S., Gu M.B. (2015). Development of highly sensitive aptamers to metabolite X for an aptamer-based diagnostic system. Abstr. Pap. Am. Chem. Soc..

[8] Wilson D.S., Szostak J.W. (1999). In vitro selection of functional nucleic acids. Annu. Rev. Biochem..

[9] Tuerk C., Gold L. (1990). Systematic evolution of ligands by exponential enrichment: RNA ligands to bacteriophage T4 DNA polymerase. Science.

[10] Mayer G. (2009). The Chemical Biology of Aptamers. Angew. Chem. Int. Ed..

[11] Wolter O., Mayer G. (2017). Aptamers as valuable molecular tools in neurosciences. J. Neurosci..

[12] Wang L., Lyu S.X., Gu G.Y., Bolten S. (2020). Selection of aptamers targeted to food-borne pathogenic bacteria *Vibrio parahaemolyticus*. Food Sci. Nutr..

[13] Wang L.J., Wang R.H., Wei H., Li Y.B. (2018). Selection of aptamers against pathogenic bacteria and their diagnostics application. World J. Microbiol. Biotechnol..

[14] Reddy P.M., Chang K.C., Liu Z.J., Chen C.T., Ho Y.P. (2014). Functionalized magnetic iron oxide (Fe_3_O_4_) nanoparticles for capturing gram-positive and gram-negative bacteria. J. Biomed. Nanotechnol..

[15] Bruno J.G., Richarte A.M. (2016). Development and characterization of an enzyme-linked DNA aptamer-magnetic bead-based assay for human IGF-I in serum. Microchem. J..

[16] Bruno J.G., Carrillo M.P., Phillips T., Edge A. (2011). Discrimination of recombinant from natural human growth hormone using DNA aptamers. J. Biomol. Tech..

[17] Shin H.S., Gedi V., Kim J.K., Lee D.K. (2019). Detection of Gram-negative bacterial outer membrane vesicles using DNA aptamers. Sci. Rep..

[18] Lonne M., Bolten S., Lavrentieva A., Stahl F., Scheper T., Walter J.G. (2015). Development of an aptamer-based affinity purification method for vascular endothelial growth factor. Biotechnol. Rep..

[19] Vergara-Barberan M., Lerma-Garciia M.J., Moga A., Carrasco-Correa E.J., Martinez-Perez-Cejuela H., Beneito-Cambra M., Simo-Alfonso E.F., Herrero-Martinez J.M. (2021). Recent advances in aptamer-based miniaturized extraction approaches in food analysis. TrAC Trend Anal. Chem..

[20] Vishwakarma A., Lal R., Ramya M. (2021). Aptamer-based approaches for the detection of waterborne pathogens. Int. Microbiol..

[21] Ho Y.-P., Reddy P.M. (2011). Advances in mass spectrometry for the identification of pathogens. Mass Spectrom. Rev..

[22] Ho Y.-P., Reddy P.M. (2010). Identification of pathogens by mass spectrometry. Clin. Chem..

[23] Fang J.S., Dorrestein P.C. (2014). Emerging mass spectrometry techniques for the direct analysis of microbial colonies. Curr. Opin. Microbiol..

[24] Liu C.W., Li B.N., Liu C., Li M., Zhou Z. (2021). Analysis of single-cell microbial mass spectra profiles from single-particle aerosol mass spectrometry. Rapid Commun. Mass. Spectrom..

[25] Giebel R., Worden C., Rust S.M., Kleinheinz G.T., Robbins M., Sandrin T.R., Allen I.L., Sima S., Geoffrey M.G. (2010). Microbial fingerprinting using matrix-assisted laser desorption ionization time-of-flight mass spectrometry (MALDI-TOF MS): Applications and challenges. Advances in Applied Microbiology.

[26] Sauer S., Kliem M. (2010). Mass spectrometry tools for the classification and identification of bacteria. Nat. Rev. Microbiol..

[27] Everley R.A., Mott T.M., Toney D.M., Croley T.R. (2009). Characterization of *Clostridium* species utilizing liquid chromatography/mass spectrometry of intact proteins. J. Microbiol. Methods.

[28] Edwards-Jones V., Claydon M.A., Evason D.J., Walker J., Fox A.J., Gordon D.B. (2000). Rapid discrimination between methicillin-sensitive and methicillin-resistant *Staphylococcus aureus* by intact cell mass spectrometry. J. Med. Microbiol..

[29] Walker J., Fox A.J., Edwards-Jones V., Gordon D.B. (2002). Intact cell mass spectrometry (ICMS) used to type methicillin-resistant *Staphylococcus aureus*: Media effects and inter-laboratory reproducibility. J. Microbiol. Methods.

[30] Demirev P.A., Fenselau C. (2008). Mass spectrometry for rapid characterization of microorganisms. Annu. Rev. Anal. Chem..

[31] Dworzanski J.P., Snyder A.P. (2005). Classification and identification of bacteria using mass spectrometry-based proteomics. Expert Rev. Proteom..

[32] VerBerkmoes N.C., Connelly H.M., Pan C., Hettich R.L. (2004). Mass spectrometric approaches for characterizing bacterial proteomes. Expert Rev. Proteom..

[33] Sauget M., Valot B., Bertrand X., Hocquet D. (2017). Can MALDI-TOF Mass spectrometry reasonably type bacteria?. Trends Microbiol..

[34] Abdel-Rahman M., Azab M.S., Meibed M., El-Kholy A., Elmetwalli A.W. (2020). Assessment of matrix-assisted laser desorption/ionization time-of-flight mass spectrometry (MALDI-TOF- MS) for accurate bacterial identification in clinical labs. Am. J. Clin. Pathol..

[35] Guo L., Ye L., Zhao Q., Ma Y., Yang J., Luo Y. (2014). Comparative study of MALDI-TOF MS and VITEK 2 in bacteria identification. J. Thorac. Dis..

[36] Liu K.-K., Chen M.-F., Chen P.-Y., Lee T.J.F., Cheng C.-L., Chang C.-C., Ho Y.-P., Chao J.-I. (2008). Alpha-bungarotoxin binding to target cell in a developing visual system by carboxylated nanodiamond. Nanotechnology.

[37] Xu X., Liu G., Huang X., Li L., Lin H., Xu D. (2020). MALDI-TOF MS-based identification of bacteria and a survey of fresh vegetables with pathogenic bacteria in Beijing, China. Food Biosci..

[38] Kuo F.Y., Lin W.L., Chen Y.C. (2016). Affinity capture using peptide-functionalized magnetic nanoparticles to target *Staphylococcus aureus*. Nanoscale.

[39] Chan P.H., Chen Y.C. (2012). Human Serum albumin stabilized gold nanoclusters as selective luminescent probes for *Staphylococcus aureus* and methicillin-resistant *Staphylococcus aureus*. Anal. Chem..

[40] Turek D., Van Simaeys D., Johnson J., Ocsoy I., Tan W. (2013). Molecular recognition of live methicillin-resistant *Staphylococcus aureus* cells using DNA aptamers. World J. Transl. Med..

