# Supplementary material for: Selective Capture and Identification of Methicillin-Resistant Staphylococcus aureus by Combining Aptamer-Modified Magnetic Nanoparticles and Mass Spectrometry
Source: Int J Mol Sci. 2021 Jun 18;22(12):6571. doi: 10.3390/ijms22126571 (PMC8234742; doi:10.3390/ijms22126571)
Supplement: Supplementary file 1 [file ijms-22-06571-s001.zip › ijms-1256842-supplementary.pdf]

SUPPORTING INFORMATION

Selective capture and identification of methicillin-resistant *Staphylococcus aureus* by combining aptamer-modified magnetic nanoparticles and mass spectrometry

1. Yu-Chen Liu, [1] Katragunta Kumar,[1] Cheng-Hsiu Wu,[1] Kai-Chih Chang,[2] Cheng-Kang Chiang[1] and Yen-Peng Ho[1*]

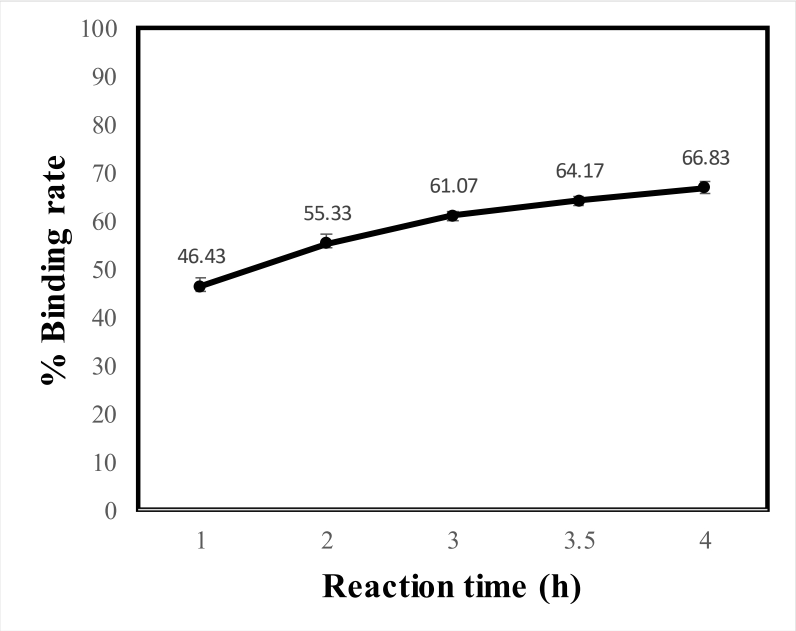

Figure S1. The binding rate vs. time of reaction between PAMNPs and aptamers.

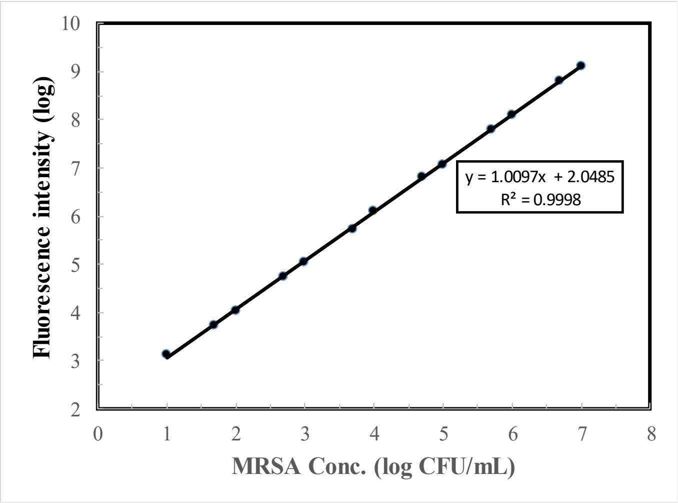

Figure 2S. Calibration curve of fluorescence vs. FITC-labeled MRSA concentration.

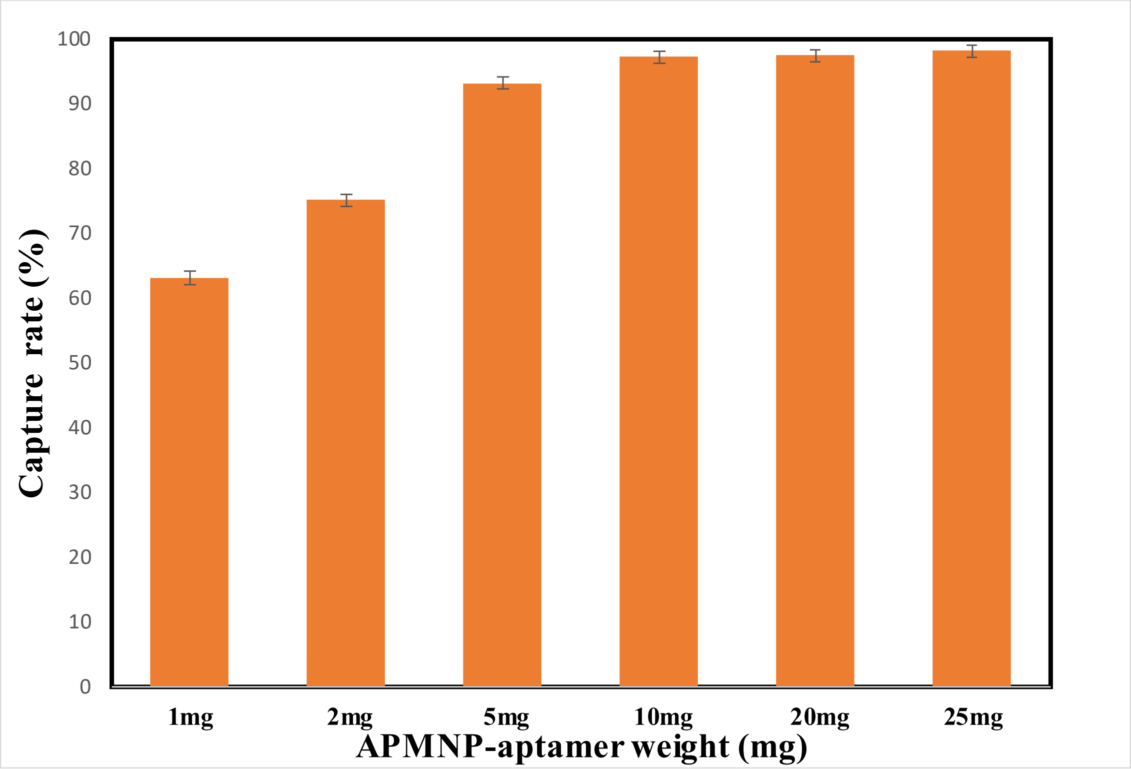

Figure 3S. Plot of MRSA capture rate vs. the amount of the modified nanoparticles in 1 mL of PBS (pH 7.5, 37°C) with 1h incubation time.
